# Monoclonal Antibodies in Alzheimer's Disease: Advances, Challenges, and Insights from a Narrative Review

**DOI:** 10.1002/alz70858_104583

**Published:** 2025-12-25

**Authors:** Gabriela Bezerra Sorato, Jessica Vargas Lopes, Ana Carolina Gomes Petry, Daniel Pasini Gonçalves, Isabela Alicia Fink, Maria Fernanda Peruci Felippe, Laura Carolina Nardi Motta, Matias Pinheiro de Macêdo Neto, Vitor Ritt Xavier, Ana Carolina Sgarioni Viapiana

**Affiliations:** ^1^ Fundação Universidade Federal de Ciências da Saúde de Porto Alegre, Porto Alegre, Rio Grande do Sul, Brazil; ^2^ Universidade Luterana do Brasil, Canoas, Rio Grande do Sul, Brazil

## Abstract

**Background:**

Alzheimer's disease (AD) is the leading cause of dementia among the elderly. Although advances in understanding its pathophysiology have led to the development of therapies, current pharmacological treatments mainly focus on symptom management and do not significantly alter disease progression. Recently, anti‐amyloid monoclonal antibodies (mAbs) have emerged as promising therapies, directly targeting beta‐amyloid plaques. However, the efficacy and safety of these therapies remain debated. This study reviews the current literature on the use of mAbs in AD.

**Method:**

A narrative literature review was conducted using articles published since 2019 and retrieved from PubMed/MEDLINE. The search terms included “*Alzheimer's disease*”, “*therapy*”, “*monoclonal antibodies*” and “*novel*”.

**Result:**

From 108 articles, four studies were included. These studies indicate that mAbs represent a significant therapeutic advance in AD, focusing on two primary therapies: aducanumab and lecanemab. Both demonstrated efficacy in reducing beta‐amyloid plaques (Ramanan VK, et al, 2023; Day GS et al, 2022) and slowing cognitive and functional decline, particularly in patients at the early stages of AD, with reductions in the cognitive decline of 25% (Ramanan VK et al, 2023) and 30% (Cummings J et al, 2023). However, the clinical benefits of these therapies remain contentious due to serious adverse effects, notably amyloid‐related imaging abnormalities, reported in 21.3% (Ramanan VK et al, 2023) to 40% (Day GS et al, 2022) of patients. Additionally, donanemab has shown the ability to remove up to 86% of cerebral amyloid; however, concerns about its safety and the statistical validity of its results (Hoilund‐Carlsen PF et al, 2023) have been raised. All treatments necessitate a robust diagnostic and monitoring infrastructure and entail high costs, which limits accessibility.

**Conclusion:**

Our analysis of studies on mAb therapy for AD concluded that these new therapies significantly impact the reduction of amyloid plaques and the slowing of cognitive decline in the early stages of the disease. Nevertheless, these alternative interventions present new risks and still need to prove their clinical applicability in more robust studies, particularly regarding safety, infrastructure, and costs. Therefore, further research should aim to address these challenges in the fight against AD using mAbs.

